# Neurophysiological modeling of bladder afferent activity in the rat overactive bladder model

**DOI:** 10.1007/s12576-015-0370-y

**Published:** 2015-03-18

**Authors:** Mahipal Choudhary, Els van Asselt, Ron van Mastrigt, Francesco Clavica

**Affiliations:** Department of Urology, Sector FURORE, Erasmus MC, Room EE1630, Dr. Molewaterplein 50, 3015 GE Rotterdam, The Netherlands

**Keywords:** Overactive bladder, Detrusor overactivity, Afferent activity, Whole nerve recordings, Rat, Mathematical modeling

## Abstract

The overactive bladder (OAB) is a syndrome-based urinary dysfunction characterized by “urgency, with or without urge incontinence, usually with frequency and nocturia”. Earlier we developed a mathematical model of bladder nerve activity during voiding in anesthetized rats and found that the nerve activity in the relaxation phase of voiding contractions was all afferent. In the present study, we applied this mathematical model to an acetic acid (AA) rat model of bladder overactivity to study the sensitivity of afferent fibers in intact nerves to bladder pressure and volume changes. The afferent activity in the filling phase and the slope, i.e., the sensitivity of the afferent fibers to pressure changes in the post-void relaxation phase, were found to be significantly higher in AA than in saline measurements, while the offset (nerve activity at pressure ~0) and maximum pressure were comparable. We have thus shown, for the first time, that the sensitivity of afferent fibers in the OAB can be studied without cutting nerves or preparation of single fibers. We conclude that bladder overactivity induced by AA in rats is neurogenic in origin and is caused by increased sensitivity of afferent sensors in the bladder wall.

## Introduction

The International Continence Society (ICS) defines the ‘overactive bladder’ (OAB) as a “symptom syndrome suggestive of lower urinary tract dysfunction”. The OAB is characterized by “urgency, with or without urge incontinence, usually with frequency and nocturia” [1]. Despite years of research, the pathophysiology of the OAB syndrome is still unknown. Several theories have been proposed to explain the mechanisms behind OAB [2]. The most prevalent — the neurogenic theory — relates the OAB syndrome to a dysfunction of brain and/or spinal cord as well as to increased sensitivity of bladder afferent sensors. Most researchers classify afferent fibers into two broad categories: the Aδ fibers which respond to bladder stretch and/or filling, pressure, and stress [3] under normal conditions, and the C fibers responding to noxious chemical or thermal stimuli [4, 5]. Alterations in the sensitivity of any/both of these bladder sensors/fibers can lead to bladder overactivity.

To study the change in bladder sensation in OAB, several animal models have been proposed [6, 7]. The acetic acid (AA) rat model of bladder overactivity, i.e., evoking voiding contractions in anesthetized rats by filling the bladder with AA has been reported to simulate, to some extent, the human OAB pathophysiology. It has shown an increase in the frequency of spontaneous (involuntary) contractions of the bladder [8, 9], an increased residual volume and a decreased inter-voiding interval [9, 10].

To measure the afferent activity in bladder nerves, various techniques have been proposed [11–14]. Among the popular ones are single fiber recordings and measurements after a central/peripheral transection of bladder nerves [14]. Apart from the difficulties involved in the preparation of single fibers and transection of nerves, they pose several other complications. Firstly, a single fiber may not represent the whole nerve. Afferent measurements obtained from single fibers only show the frequency of firing and fail to take into account the interaction among the unitary spikes [15]. Measurement and modeling of these interactions and of the recruitment of new fibers when the bladder is exposed to external stimuli are necessary for the development of implantable electrical devices for neural control of micturition, e.g., in spinal cord injured patients. Additionally, transection of the pelvic nerve has been reported to affect the afferent, sympathetic and parasympathetic fibers’ neural activity, hence affecting the complete physiological voiding cycle [16].

Previously, in order to study the afferent nerve activity in intact, mixed (i.e., containing both afferent and efferent fibers) postganglionic bladder nerves, we developed a mathematical model which enabled the differentiation of total measured nerve activity into afferent and efferent activity without cutting the nerve or preparing single fibers [11]. In short, lesion experiments were done in which bladder pressure and nerve activity were recorded before and after a central cut of the bladder nerve. Afferent activity was linearly related to the recorded pressure. Similar relationships were found when afferent activity was recorded from cut nerves and when it was estimated from intact nerve experiments. Additionally, peripheral cutting and pelvic afferent nerve stimulation showed that when electrical stimulation of the pelvic nerve was suddenly aborted, resulting in a sudden stop of efferent nerve activity, a decrease in bladder pressure was found that was very similar to the decrease that occurred during voiding contractions. This model was developed and validated in saline (physiologically normal) bladder filling experiments. Using this model, the slope of the linear dependence of afferent activity on bladder pressure (which is a measure for the sensitivity of the afferent fibers/sensors for pressure change) can be estimated.

In the present study, we applied this mathematical model to the AA rat model of bladder overactivity to study the changes in sensitivity of afferent nerve fibers in intact nerves, i.e., without transection or separation of single fibers.

## Materials and methods

### Experimental procedures

All the laboratory and animal experimental procedures described in this study were approved by the local Erasmus MC Animal Experiment Committee. Eighteen male Wistar rats (mean weight 420 ± 44 g) were anesthetized with urethane (1 g/kg) intraperitoneally. An abdominal incision was made, the postganglionic bladder nerves close to the bladder (branches of the pelvic nerve) were carefully dissected and thin non-absorbable, sterile sutures were tied around the identified nerves. Warm saline (0.9 % NaCl) was poured into the abdomen to prevent drying out of the organs during surgery.

**Fig. 1:**
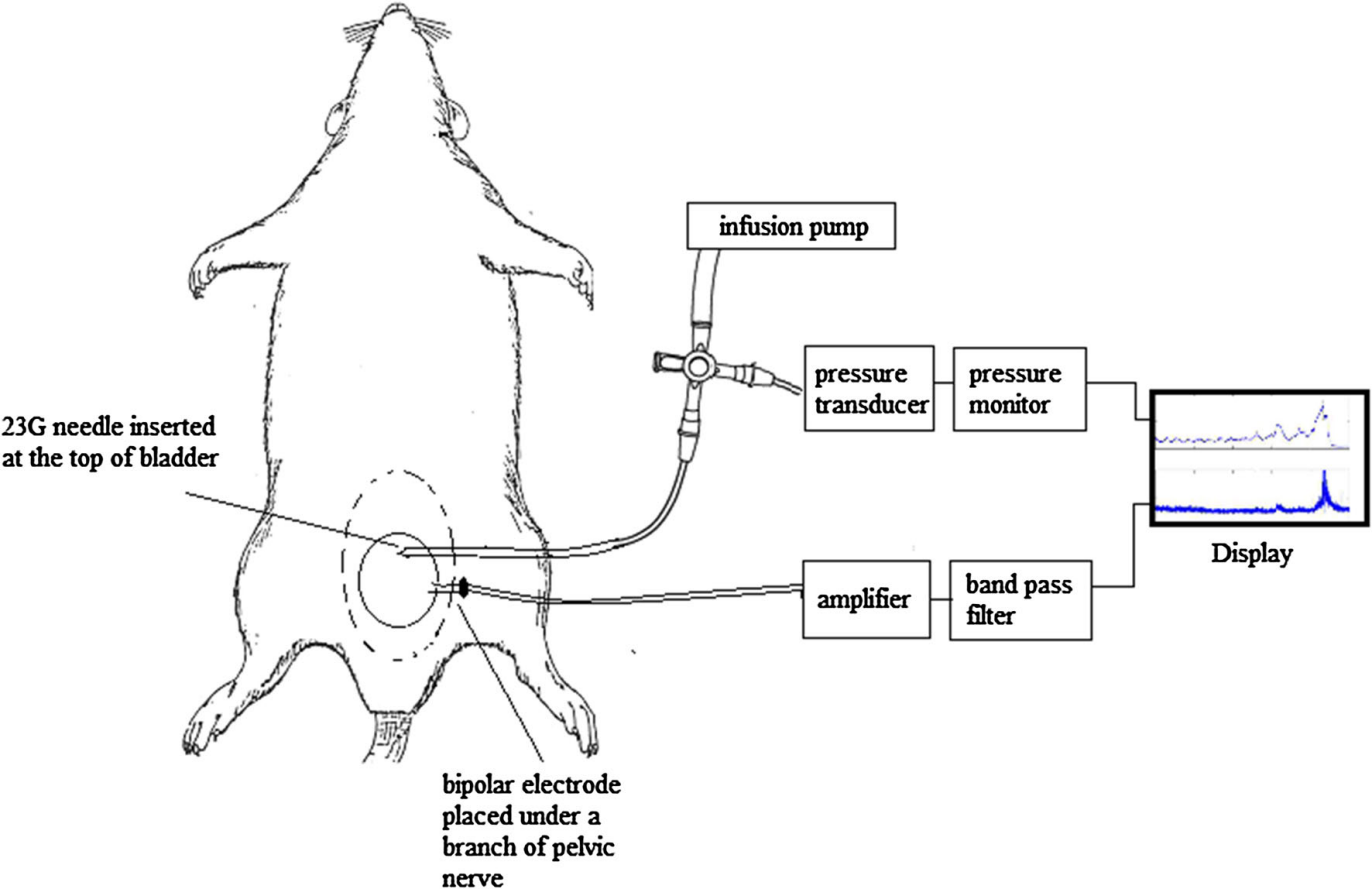
Experimental set-up. After exposing the abdominal cavity, a 23G needle was inserted at the top of the bladder for filling and pressure measurement. The other end of the needle was attached to a disposable pressure transducer and an infusion pump using a 2-way connector. Custom-made bipolar electrodes consisting of two thin platinum–iridium hook shaped wires separated by a distance of 0.5–1 mm were placed under one of the branches of the pelvic nerve using a micromanipulator. The recorded nerve signal was then amplified and band pass filtered

After the surgical procedures, the saline was removed and the pelvic cavity was kept moist by warm paraffin oil throughout the electrophysiological measurements. Bladder pressure measurement and bladder filling (0.05 ml/min) were done by inserting a 23G needle at the top of the bladder. The other end of the needle was attached to a disposable pressure transducer and an infusion pump using a 2-way connector. Pressure was measured using a Statham SPI400 blood pressure monitor. The bladder was repeatedly filled with saline or 0.5 % AA until a voiding contraction occurred or for a maximum duration of 20 min (up to ~1 ml), whichever happened first. Custom-made bipolar electrodes consisting of two thin (diameter 0.1 mm) platinum-iridium hook-shaped wires separated by a distance of 0.5–1 mm were used for the nerve signal recordings. The electrode was mounted on a micromanipulator and one of the identified nerves was carefully placed on the wires. A brief illustration of the experimental set-up is shown in Fig. 1. In 4 of the 18 rats, a left side postganglionic pelvic nerve branch was crushed between the major pelvic ganglion and the electrode, to eliminate efferent nerve signals and record afferent activity only. The pressure transducer was calibrated using a column of water before the start of each experiment. The nerve signal was calibrated using a 1-µV (500 Hz) sinusoidal test signal. The rats were euthanized at the end of the experiment with an overdose of KCl, injected into the heart.

### Data acquisition and storage

The nerve activity was amplified by a DISA 15C01 EMG amplifier (amplification range: 100–200,000) and band-pass filtered with a Krohn-Hite 3944 filter (Bessel, 4th order, 200–2000 Hz). Bladder pressure and nerve activity were displayed in real-time on a computer screen using a custom-written LabVIEW^®^ (National Instruments, USA) program.

In the first few rats the pressure and nerve signals were recorded for periods of 30 s, which contained at least one voiding contraction. The computer program was later optimized to record intervals of 5 min.

### Signal processing

Nerve activity and pressure signals were sampled at 25 kHz and 25 Hz, respectively. Measurements with movement artifacts due to catheter displacement, air bubbles in the set-up or movement of the rat, etc., were excluded from analysis. These artifacts were observed during data acquisition and were seen as random, sharp high amplitude peaks in the pressure curve.

Recorded signals were processed and analyzed with a custom-written MATLAB^®^ (Mathworks, USA) program. In the first step, the nerve signal was rectified and averaged [17, 18] by taking the mean of each 1,000 samples, effectively reducing a 1 s interval to 25 data samples.

The quality of the nerve activity recordings was assessed by the signal to noise ratio (SNR) of the averaged signal [3, 11]. Assuming that the lowest values in the averaged nerve activity recording represented baseline nerve activity and the highest values are caused by nerve action potentials, the SNR was estimated as the difference between the means of the ten highest and the ten lowest average nerve activity values divided by the mean of the ten lowest average values.

To allow for a comparison of results between animals, the averaged nerve signal was normalized by dividing it by the maximum afferent activity, calculated as the mean of the ten highest average nerve activity values. Similarly, pressure was normalized by dividing by the maximum pressure.

**Fig. 2:**
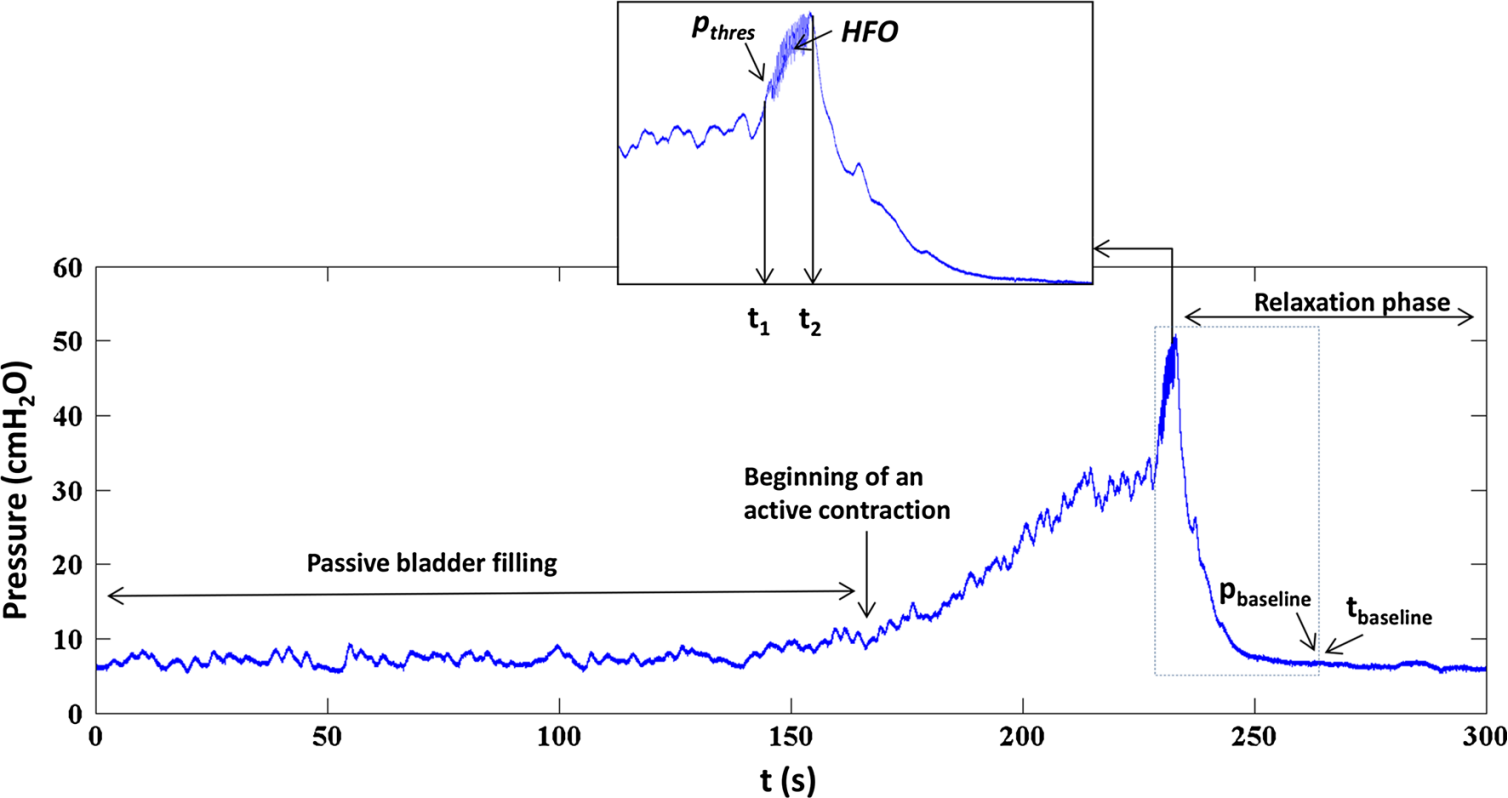
Pressure development during bladder filling. Bladder pressure recorded during one complete voiding cycle of a rat. *t* = 0 represents the start of bladder filling, at *t*_1_ − *t*_2_ voiding takes place and *t* > *t*_2_ represents the relaxation phase

The whole micturition cycle shown in Fig. 2 was divided into three phases:

1. The filling phase (*t* < *t*_1_)
  The filling phase was further divided into two sub-phases:
  Passive filling phase: from start of filling (at *t* = 0) when the bladder was compliant and the pressure was relatively low.
  Active contraction: the part of the pressure curve where the bladder contracts (marked as beginning of an active contraction) and the pressure increased till *t*_1_, which represents the start of voiding. The pressure at *t*_1_ is the maximum pressure immediately before the start of flow and has been termed ‘pressure threshold’ in the literature [7, 19].
2. The voiding phase (*t*_1_ − *t*_2_)

During this phase the actual voiding takes place. A typical rat voiding contraction is associated with high frequency oscillations (HFO), caused by rapid contractions of the urethral sphincter [7]. The HFO were visually identified in the pressure recording (Fig. 2, *t*_1_ − *t*_2_). In Fig. 2, *t*_1_ is the time at the start of HFO: the pressure at *t*_1_ has been termed the threshold pressure (*p*_thres_) [7]. *t*_2_ is the time at maximum pressure, after cessation of the HFO. The start of flow is characterized by the expulsion of urine droplets, which is synchronous with the initiation of HFO, both of which can be observed visually. The period *t*_1_ − *t*_2_ was excluded from analysis because of movement artifacts in the recorded nerve and pressure signal.

3. The relaxation phase (*t* > *t*_2_)

In this phase the bladder relaxed isovolumetrically and pressure decreased to baseline. The baseline pressure (*p*_baseline_) was determined by a computer algorithm that divided the pressure signal (*t* > *t*_2_) into successive windows of 1 s. The mean pressure in each window was compared to that in the next window. When the difference between the means of two consecutive windows was <2 cmH_2_O, the mean pressure in the first window was taken as the baseline.

### Modeling

#### Step 1: Test of absence of efferent contribution in the relaxation phase

We have earlier shown in saline filling experiments that nerve activity after a voiding contraction is all afferent. To confirm that this is also the case in the AA measurements, we calculated the time constant of pressure decay in the interval *t*_2_ − *t*_baseline_ and compared it between saline and AA measurements.

The time constant of exponential decay was calculated by fitting:

$$p\left( t \right) = Ae^{ - t/\tau },$$

where p(*t*) is the pressure in the interval *t*_2_ − *t*_baseline_, *A* is constant and *τ* is the time constant.

To provide additional support for the assumption that there is no efferent activity in the relaxation phase, only afferent nerve activity was measured (see “Experimental procedures” section) and the slope of afferent nerve activity and pressure in the relaxation phase in saline and AA measurements was calculated. The slope in these crushed nerve experiments was compared with that of afferent nerve activity and pressure in the relaxation phase in intact nerve experiments (see Step 2).

#### Step 2: Derivation of afferent activity in the filling phase

The averaged nerve signal in the relaxation phase *t*_2_ − *t*_baseline_ was smoothed with a first-order Savitzky-Golay FIR smoothing filter and a linear regression model was fitted to the nerve activity-pressure data using the MATLAB^®^ function ‘polyfit’ to calculate slope and offset:

$${\text{NA}}(t) = m\star p(t) + {\text{NA}}_{0},$$

where *m* is the slope of the pressure-nerve activity data in the interval *t*_2_ − *t*_baseline_, NA_0_ is the offset which represents the baseline nerve activity, *p*(*t*) is the pressure during the relaxation phase *t*_2_ − *t*_baseline_ and NA(*t*) is the afferent nerve activity in the interval *t*_2_ − *t*_baseline_. Once the slope and offset were known, the afferent activity in the *filling phase* was calculated using the same formula.

#### Step 3: Modeling of afferent activity and volume in the filling phase

In addition to the afferent nerve activity-pressure modeling, we also modeled the relationship between afferent activity and bladder volume in the filling phase. A linear regression model was fitted to the afferent nerve activity-volume data to calculate slope and offset, as described in Step 2. Additionally, the linear correlation coefficient (Pearson’s product-moment correlation), which measures the linear dependence of afferent activity on the bladder volume was also calculated using the MATLAB^®^ function ‘corrcoef’.

All data are presented as mean ± SD. The Mann–Whitney *U* test was performed using SPSS^®^ statistical package (version 21.0, SPSS Inc., Chicago, IL, USA) to compare groups. A *p* < 0.05 was considered significant.

## Results

### Intact nerve experiments

**Fig. 3:**
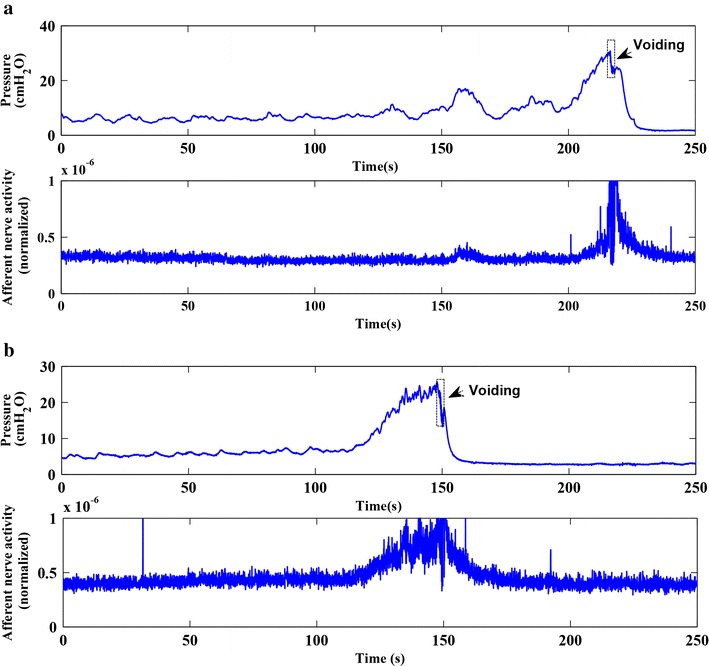
Nerve activity and bladder pressure during a voiding cycle. An example of a pressure-afferent nerve activity measurement with saline and AA filling in the same rat. The bladder filling started at *t* = 0. The *upper panel* of both **a** and **b** shows the pressure during filling phase and a typical rat voiding contraction. The *lower panel* of **a** and **b** shows the nerve activity

Postganglionic bladder nerve activity and bladder pressure were successfully measured in 10 out of 14 rats. A total of 84 measurements were recorded from these 10 rats (Table 1): 28 measurements were excluded because of artifacts, as described in the preceding section. The remaining 56 measurements had a SNR >0.5, i.e., the nerve activity was ≥50 % higher than baseline activity. For the analysis of the filling phase, an additional 21 measurements were excluded because of artifacts and parameters were calculated for 35 measurements (saline = 15, AA = 20). Figure 3 shows an example of a good pressure-nerve activity recording with saline and AA filling from the same rat. The bladder filling started at *t* = 0 and was stopped at the beginning of a voiding contraction. After the voiding contraction, both in saline and AA, the pressure declined smoothly to baseline in a similar fashion. It can be noted that similar to pressure, the nerve activity also followed a smoothly declining pattern.

**Table 1:** The number of saline and acetic acid measurements in 10 rats

Rat number	Saline		Acetic acid	
	Total	Included	Total	Included
Rat1	2	2	3	3
Rat2	7	6	6	2
Rat3	4	2	2	1
Rat4	4	2	3	3
Rat5	4	3	6	4
Rat6	2	2	3	2
Rat7	3	2	5	3
Rat8	5	1	6	4
Rat9	5	3	6	3
Rat10	4	4	4	4
Σ=	40	27	44	29

In the filling phase, the baseline afferent activity and the threshold pressure (*p*_thres_) before a voiding contraction did not differ significantly between saline and AA measurements. However, the afferent activity in the active contraction interval was significantly higher in AA as compared to saline measurements. The bladder capacity (filled volume at which voiding occurred) was reduced by 31 % in AA measurements (Table 2). The slope of afferent nerve activity-volume was significantly higher in AA measurements than in saline measurements. The offset did not differ significantly between the two groups. The correlation coefficient of the linear dependence of afferent activity on bladder volume was also significantly higher in AA measurements (Table 2).

**Table 2:**
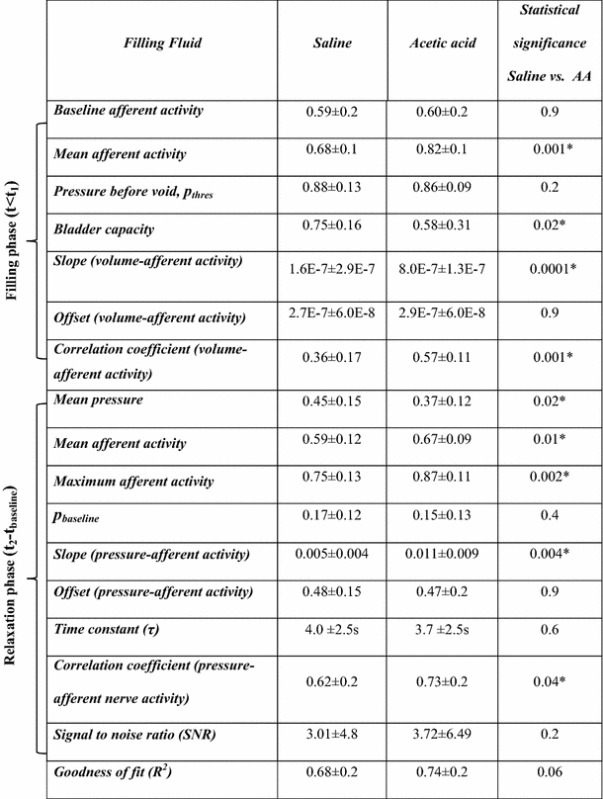
Mean ± SD of the estimated normalized parameters in saline and acetic acid measurements, *n* = 35 (saline = 15, AA = 20) for filling phase parameters and *n* = 56 (saline = 29, AA = 27) for relaxation phase parameters. Mann–Whitney test (* *p* < 0.05)

**Fig. 4:**
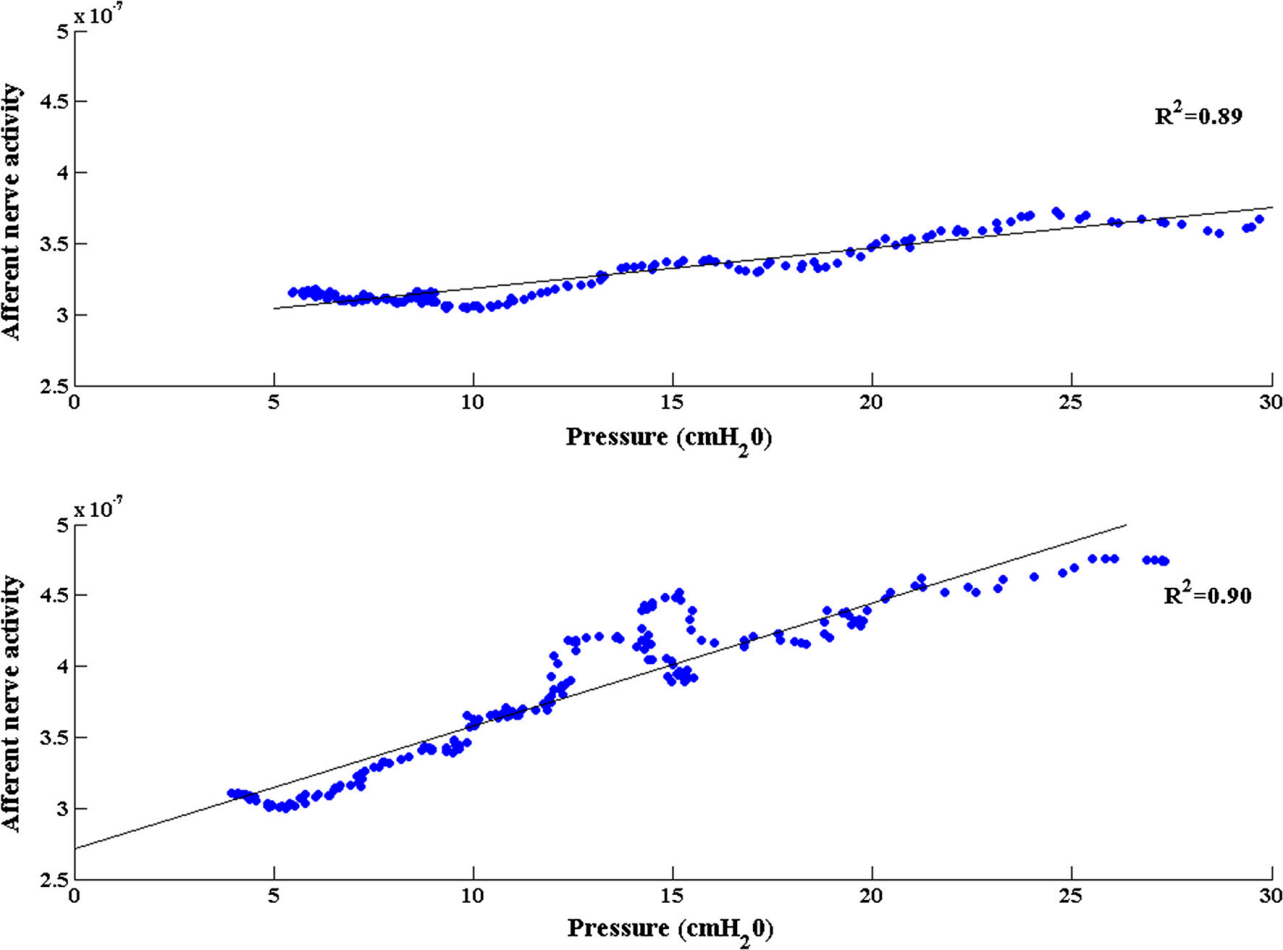
Linear relationship between afferent activity and bladder pressure. A linear polynomial fit of afferent nerve activity as a function of bladder pressure recorded during the relaxation phase of a voiding contraction in the same rat after saline (*upper panel*) and acetic acid (*lower panel*) filling. *Dots* represent the measured afferent activity and the *solid line* represents the fitted model

In the post-void relaxation phase, the time constant of exponential pressure decay (*τ*) in the interval *t*_2_ − *t*_baseline_ was not significantly different between AA and saline measurements. The average afferent activity, the maximum afferent activity and the mean value of the normalized slope in the interval *t*_2_ − *t*_baseline_ were significantly higher in AA than in saline measurements, whereas the mean value of pressure (*t*_2_ − *t*_baseline_) was found to be higher in saline measurements. The baseline afferent activity and baseline pressure (*p*_baseline_) after the voiding contraction did not differ significantly (Table 2). The correlation coefficient (Pearson’s product-moment correlation), which is a measure of the linear dependence of nerve activity on bladder pressure, was significantly higher in AA (Table 2), whereas the SNR and the goodness of fit (*R* ^2^), which describes how well the model fitted the data, did not differ significantly. An example of a fitted line in a saline and an AA measurement is shown in Fig. 4.

### Crushed nerve experiments

In the crushed nerve experiments (4 rats), the slope of afferent nerve activity and pressure was higher in AA (*n* = 10) as compared to saline (*n* = 9) measurements. After removal of 2 obvious outliers from the saline measurements, the slope in the AA measurements was twice (0.007 ± 0.005) that of the saline (0.003 ± 0.002) measurements (*p* = 0.06), similar to the change in slope in intact nerve experiments. The offset did not differ significantly between saline (2.3E−7 ± 5.0E−8) and AA (2.1E−7 ± 3.0E−8) measurements (*p* = 0.5). The threshold pressure between saline (33.0 ± 6.0 cmH_2_O) and that in AA (31.0 ± 7.5 cmH_2_O) was also found to be comparable (*p* = 0.5).

## Discussion

In this study we quantitatively compared the sensitivity of afferent nerve fibers in a rat OAB model with that in normal rats without cutting nerves or preparing single fibers. Several models have been reported in the literature for studying the afferent mechanisms of the urinary bladder. Sadananda et al. [13] proposed a decerebrated arterially perfused rat in situ model (DAPR) that allows the study of micturition in rats. The model offers the advantage of studying the bladder neurophysiology without the need for anesthesia and a faster recording set-up. However, the authors reported a 4-h duration of viability, which imposes a limitation to experiments requiring more time. Additionally, the nerves were transected in order to record the afferent nerve activity. The cutting of a nerve has been reported to disrupt the protein supply to the nerve, leading to nervous tissue death, which would be disadvantageous for recording nerve activity for longer periods of time [20]. Zvara et al. [12] presented an in situ model where afferent recordings were made by transecting bladder nerves in non-anesthetized mice. The model also has the advantage of afferent nerve activity measurements without the need for anesthesia. However, electrophysiological recordings in freely moving animals suffer from movement artifacts. Additionally once the nerve is enclosed and the abdominal cavity is sutured, it is very difficult to manipulate/modify the nerve-electrode interface. The model presented in the current study offers the advantage of minimal surgical procedures and enables recordings from intact nerves with minimal damage. Although the effect of anesthesia on neurophysiology cannot be denied, it has been reported to spare micturition [21].

The mathematical model used in the current study assumes that the nerve activity measured from an intact, multifiber rat bladder nerve in the relaxation phase of a voiding contraction is purely afferent and that it depends linearly on the bladder pressure. In an earlier study in anesthetized rats of the same age, weight and gender, these hypotheses were verified by proximal cutting of bladder nerves and electrical stimulation of the pelvic nerve [3, 11]. In the present study the mathematical model was applied to an OAB rat model. It was found that the correlation between pressure and nerve activity was even better in AA measurements than in saline fillings, implying that the linear relationship between pressure and afferent nerve activity holds true for the overactive bladder (Fig. 4). The coefficients of that linear relationship, derived from the post-void relaxation phase, can be used to estimate the afferent activity in the filling phase. Although this finding has no direct clinical implication, it could be useful in closed-loop implantable devices for detecting changes in afferent nerve activity based on pressure recordings and electrically inhibiting bladder contractions. We also modeled the relationship between afferent nerve activity and bladder volume in the filling phase, and it was found that the afferent nerve activity (in both saline and AA) correlated better with pressure than with volume. This can be attributed to the compliance of the bladder during filling: while the bladder volume increases constantly, the bladder pressure and the afferent nerve activity remain relatively unchanged (or very low), till a certain threshold is reached, at which a voiding contraction begins and both pressure and afferent activity rise simultaneously.

To verify the absence of efferent activity in the relaxation phase in intact nerve measurements, we calculated the time constant of the isovolumetric pressure decay after a voiding contraction. It was assumed that in the absence of any efferent activation, the bladder relaxes passively and the time constant would be comparable in saline and AA measurements. Conversely, any efferent activity would result in a contraction or relaxation of the bladder, which in turn would result in a larger or smaller time constant. In Fig. 3, it can be seen in both saline and AA filling pressure curves that, after a voiding contraction, the bladder relaxed passively, and the pressure declined smoothly to baseline and stayed low. Conversely, in the filling phase, the bladder showed continuous non-voiding contractions (pressure transients with an amplitude 0.5–3 cmH_2_O [22]), which have been reported to be associated with pelvic efferent nerve discharge during the continence process [23]. In addition to our earlier findings that there is no efferent activity after a voiding contraction in saline measurement [11], the slope of the relationship between pressure and afferent nerve activity in intact and crushed nerve experiments showed a similar relationship in saline and AA measurements. The similar pattern of pressure decrease after a voiding and the comparable time constants in saline and AA fillings indicate that there is no (or insignificant) efferent activity after a voiding contraction, either in saline or in AA measurements.

Bladder overactivity on instillation of 0.5 % AA was assessed by various urodynamic parameters. To provide reassurance that in our model we have produced sensitization like that previously reported, we measured the urinary bladder capacity. We found a 31 % reduction of capacity in acetic acid instillation measurements as compared to saline filling, which is in line with other studies reporting a relevant reduction of bladder capacity with acetic acid [9, 24]. Figure 3 shows a good example of the difference between bladder capacities in saline and AA in the same rat, where it can be seen that due to reduced bladder capacity, voiding occurred earlier (at lower volume) in AA. The threshold pressure just before a voiding contraction was not significantly different between acetic acid and saline filling, which is also supported by other studies [9, 25, 26]. This implies that compared to saline fillings, there was higher afferent activity and comparable bladder pressure in acetic acid measurements at a significantly lower volume. AA of 0.2–0.5 % has been reported to induce bladder overactivity in rats without significant morphological alterations [27]. In this study, repeated infusion with saline was followed by repeated infusion with AA in order to compare and validate our results with the literature. However, in a different study (unpublished), where a ‘saline-AA-saline’ filling routine was followed, we found that the bladder capacity was significantly reduced by AA and in consequent fillings with saline the bladder capacity did not return to its original value within the time scheme (60–90 min) in which these experiments were done. This indicated that although AA does not cause significant damage to bladder morphology, it does cause a ‘carry on’ effect on bladder afferent sensitization. The AA rat model of OAB in this study closely resembles the rat model of cystitis [28, 29] with overlapping symptoms such as an increased urgency, frequency and nocturia. However, there might still be a different mechanism of action behind OAB and cystitis.

We found higher afferent activity both in the filling phase and the relaxation phase when the bladder was filled with acetic acid than when it was filled with saline. This is in accordance with other models of bladder overactivity [12, 30]. The multifiber whole nerve activity recorded in our study does not allow the distinction of myelinated and unmyelinated fibers. However, the significant changes in afferent nerve activity in response to AA filling indicated that whole nerve recordings do reflect (pathophysiological) changes in nerve activity. The slope of the linear dependence of afferent nerve activity on bladder pressure, which defines the sensitivity of the afferent fibers/sensors for pressure changes, was significantly higher in acetic acid measurements than in saline measurements. This implies a higher sensitivity (response) of afferent fibers under overactive bladder conditions at a given pressure. The increased afferent nerve activity indicates an underlying neurogenic mechanism: the bladder overactivity induced by acetic acid could be due to a direct sensitization of afferent nerve endings by AA. However, it could also be due to an effect of acetic acid on the bladder urothelium, which in turn signals strongly to the afferents, thus causing an increased sensitivity. The relationship between afferent nerve activity and pressure was studied at a filling rate of 50 µl/min which is within the practical range of physiological filling rates [14]. In previous studies it was found that at supraphysiological (200 µl/min and above) filling rates the afferent response was significantly reduced [14]. The baseline nerve activity, both in the filling phase and the relaxation phase did not differ significantly between saline and acetic acid measurements. This implies that the sensitivity of afferent fibers at very low pressure (bladder empty) was not affected by bladder overactivity induced by acetic acid. The calculated baseline was compared with a conventional method by recording signals after euthanizing animals, where no difference was found [4 rats, (1.01 × 10^−7^ ± 8 × 10^−10^) vs (1.008 × 10^−7^ ± 3 × 10^−10^), *p* = 0.15]. A plausible explanation for this could be that at low bladder pressure, the afferent fibers have a very low firing rate and the measured signal consists mainly of noise (~constant). The results on baseline nerve activity are in agreement with other studies in the rat [5, 31, 32], where it was reported that pelvic afferent fibers showed little or no nerve activity when the bladder was empty.

## Conclusion

We have shown that our previously proposed mathematical model is applicable to the acetic acid rat model of bladder overactivity. Using this model, the sensitivity of afferent fibers can be studied in the overactive bladder condition in intact bladder nerves without the need for cutting nerves or preparing single fibers. To our knowledge, this is the first study comparing the afferent nerve activity measured from intact mixed nerves, in a physiologically optimal condition, in normal and overactive bladders. We found that the sensitivity of afferent fibers, estimated using this model, was higher in acetic acid measurements than in saline measurements, while the maximum pressure before the voiding contraction did not differ significantly between both groups. This leads to the conclusion that the bladder overactivity induced by acetic acid in rats is neurogenic in origin and is caused by increased sensitivity of afferent sensors in the bladder wall.

## References

[1] Abrams P (2003). Describing bladder storage function: overactive bladder syndrome and detrusor overactivity. Urology.

[2] Hashim H, Abrams P (2007). Overactive bladder: an update. Curr Opin Urol.

[3] le Feber J, van Asselt E, van Mastrigt R (2004). Afferent bladder nerve activity in the rat: a mechanism for starting and stopping voiding contractions. Urol Res.

[4] Wyndaele JJ (2010). Investigating afferent nerve activity from the lower urinary tract: highlighting some basic research techniques and clinical evaluation methods. Neurourol Urodyn.

[5] Moss NG, Harrington WW, Tucker MS (1997). Pressure, volume, and chemosensitivity in afferent innervation of urinary bladder in rats. Am J Physiol.

[6] Parsons BA, Drake MJ (2011) Animal models in overactive bladder research. Handb Exp Pharmacol: 15–43 10.1007/978-3-642-16499-6_2 21290220

[7] Andersson KE, Soler R, Fullhase C (2011). Rodent models for urodynamic investigation. Neurourol Urodyn.

[8] Tai C, Shen B, Chen M, Wang J, Liu H (2011). Suppression of bladder overactivity by activation of somatic afferent nerves in the foot. BJU Int.

[9] Kakizaki H, de Groat WC (1996). Role of spinal nitric oxide in the facilitation of the micturition reflex by bladder irritation. J Urol.

[10] Mitsui T, Kakizaki H, Matsuura S, Ameda K, Yoshioka M (2001). Afferent fibers of the hypogastric nerves are involved in the facilitating effects of chemical bladder irritation in rats. J Neurophysiol.

[11] Le Feber J, Van Asselt E, Van Mastrigt R (1997). Neurophysiological modeling of voiding in rats: bladder pressure and postganglionic bladder nerve activity. Am J Physiol.

[12] Zvara P, Wright AJ, Roach K, Ursiny M, Shapiro B (2010). A non-anesthetized mouse model for recording sensory urinary bladder activity. Front Neurol.

[13] Sadananda P, Drake MJ, Paton JF, Pickering AE (2011). An exploration of the control of micturition using a novel in situ arterially perfused rat preparation. Front Neurosci.

[14] De Wachter S, De Laet K, Wyndaele JJ (2006). Does the cystometric filling rate affect the afferent bladder response pattern? A study on single fibre pelvic nerve afferents in the rat urinary bladder. Neurourol Urodyn.

[15] Dick DE, Meyer JR, Weil JV (1974). A new approach to quantitation of whole nerve bundle activity. J Appl Physiol.

[16] Vaughan CW, Satchell PM (1995). Urine storage mechanisms. Prog Neurobiol.

[17] Andresen MC, Yang M (1989). Interaction among unitary spike trains: implications for whole nerve measurements. Am J Physiol.

[18] Hopp FA, Seagard JL, Kampine JP (1986). Comparison of four methods of averaging nerve activity. Am J Physiol.

[19] Yamada A, Torimoto K, Obata K, Hirayama A, Fujimoto K (2014). Persistent overexpression of SERCA2a affects bladder functions under physiological conditions, but not in bladder outlet obstruction-induced sub-acute pathological conditions. J Physiol Sci.

[20] Gilley J, Coleman MP (2010). Endogenous Nmnat2 is an essential survival factor for maintenance of healthy axons. PLoS Biol.

[21] Khadra MH, Satchell PM, Vaughan CW (1995). Sympathetic nervous system effects on feline bladder wall compliance throughout continence. Acta Physiol Scand.

[22] Streng T, Hedlund P, Talo A, Andersson KE, Gillespie JI (2006). Phasic non-micturition contractions in the bladder of the anaesthetized and awake rat. BJU Int.

[23] Satchell P, Vaughan C (1989). Efferent pelvic nerve activity, ganglionic filtering, and the feline bladder. Am J Physiol.

[24] Matsuta Y, Mally AD, Zhang F, Shen B, Wang J (2013). Contribution of opioid and metabotropic glutamate receptor mechanisms to inhibition of bladder overactivity by tibial nerve stimulation. Am J Physiol Regul Integr Comp Physiol.

[25] Kitagawa Y, Wada M, Kanehisa T, Miyai A, Usui K (2013). JTS-653 blocks afferent nerve firing and attenuates bladder overactivity without affecting normal voiding function. J Urol.

[26] Clavica F, Choudhary M, van Asselt E, van Mastrigt R (2014) Frequency analysis of urinary bladder pre-voiding activity in normal and overactive rat detrusor. Neurourol Urodyn. doi:10.1002/nau.22664 10.1002/nau.22664 25201641

[27] Mitobe M, Inoue H, Westfall TD, Higashiyama H, Mizuyachi K (2008). A new method for producing urinary bladder hyperactivity using a non-invasive transient intravesical infusion of acetic acid in conscious rats. J Pharmacol Toxicol Methods.

[28] Aizawa N, Igawa Y, Nishizawa O, Wyndaele JJ (2011). Effects of nitric oxide on the primary bladder afferent activities of the rat with and without intravesical acrolein treatment. Eur Urol.

[29] Minagawa T, Aizawa N, Igawa Y, Wyndaele JJ (2014). The role of transient receptor potential ankyrin 1 (TRPA1) channel in activation of single unit mechanosensitive bladder afferent activities in the rat. Neurourol Urodyn.

[30] Ikeda M, Kawatani M, Maruyama T, Ishihama H (2006). Prostaglandin facilitates afferent nerve activity via EP1 receptors during urinary bladder inflammation in rats. Biomed Res.

[31] Sengupta JN, Gebhart GF (1994). Mechanosensitive properties of pelvic nerve afferent fibers innervating the urinary bladder of the rat. J Neurophysiol.

[32] van Asselt E, le Feber J, van Mastrigt R (1999). Threshold for efferent bladder nerve firing in the rat. Am J Physiol.

